# PrP^res^ deposition in the retina is a common finding of sporadic, familial and iatrogenic Creutzfeldt-Jakob diseases (CJD)

**DOI:** 10.1186/s40478-018-0582-5

**Published:** 2018-08-10

**Authors:** Masaki Takao, Hiroaki Kimura, Tetsuyuki Kitamoto, Ban Mihara

**Affiliations:** 1 Department of Neurology, Saitama International Medical Center, Saitama Medical University, 1397-1 Yamane, Hidaka, Saitama, 350-1298 Japan; 2 Department of Neurology and Brain Bank, Mihara Memorial Hospital, 366 Ohtemachi, Isesaki, Gunma 372-0006 Japan; 3 Department of Neurological Sciences, Tohoku University, Graduate School of Medicine, 2-1 Seiryo-machi, Aoba-ku, Sendai, 980-8575 Japan

**Keywords:** Prion, Creutzfeldt-Jakob disease, Retina, Immunohistochemistry

## Text

Creutzfeldt-Jakob disease (CJD) is clinically characterized by progressive dementia and neuropathologically characterized by deposits of a protease-resistant isoform of the prion protein (PrP^res^) in the central nervous system. PrP^res^ deposits in the neural retina were identified in the outer and inner plexiform layers (OPL and IPL) in a limited number of sporadic Creutzfeldt-Jakob diseases (sCJD) and two variant CJDs [1, 2]. However, the presence of PrP^res^ in the neural retina remains unknown in other types of CJDs. Therefore, we analyzed 16 prion cases from our brain bank, including sporadic, familial, and iatrogenic CJDs by using retinal sections [3].

At the time of autopsy, full permission was obtained from each patient’s next-of-kin. The posterior portion of the eye ball was removed with a scalpel, leaving the cornea and lens for funereal purposes. The following cases were available: nine cases of sCJD (MM1), two cases of sCJD (MM1 + 2, MM1 > 2), one case of sCJD (MM2), three cases of familial CJD (fCJD) (two of V180I and one of M232R), and one case of iatrogenic CJD (cadaveric dura mater graft, dCJD). We classified sCJDs based on the Parchi’s methodology [4, 5]. We also used four autopsy-confirmed neurological cases as controls (Table 1). For immunohistochemical studies to detect PrP^res^, formalin-fixed and formic acid-treated sections of the retina were immunolabeled with monoclonal antibodies specific to prion proteins 3F4 (109–112) (1:200, Biolegend, USA) and 12F10 (142–160) (1200, Bertin Bioreagent, France). The retinal sections were processed by using a Ventana Discovery automated immunostainer. Evaluations of 3F4 and 12F10-immunoreactive deposits (PrP^res^-irs) of the outer and inner plexiform layers were performed by using both antibodies in a semiquantitative manner: 0 = none, 1 = positive and scattered, 2 = positive (Table 1). PrP^res^-irs staining was weak or focal in the outer and inner nuclear layers (ONL and INL), as well as in the ganglion cell and nerve fiber layers (GCL and NFL); thus, we calculated the frequency of cases with PrP^res^-irs staining, separated on the basis of each anatomical region of the neural retina.

**Table 1:** PrP immunoreactivity of the retina in 16 cases of Creutzfeldt- Jakob disease (CJD)

Case number	AAD (y)	Duration (months)	Sex	Initial symptom	Diagnosis	Codon 129	Western blot analysis of PrP^res^	OPL		IPL	
								3F4-irs	12F10-irs	3F4-irs	12F10-irs
Case 1	72	18	F	visual acuity, color agnosia	sCJD	MM	Type 1	1	2	2	2
Case 2	67	51	F	amnesia	sCJD	MM	Type 1	1	2	1	2
Case 3	75	17	F	palilalia	sCJD	MM	Type 1	2	1	2	1
Case 4	74	6	M	metamorphopsia	sCJD	MM	Type 1	1	1	0	1
Case 5	73	2	M	amnesia	sCJD	MM	Type 1	2	2	2	2
Case 6	82	5	F	sensory disturbance	sCJD	MM	Type 1	1	2	1	2
Case 7	62	42	M	loss of motivation	sCJD	MM	Type 1	1	2	1	2
Case 8	68	8	M	visuospatial disturbance	sCJD	MM	Type 1	2	2	2	2
Case 9	85	7	F	communication disturbance	sCJD	MM	Type 1	2	2	2	2
Case 10	67	30	M	amnesia	sCJD	MM	Type 2	1	2	1	2
Case 11	62	10	F	dizziness, ataxia	sCJD	MM	Type 1 + 2	2	2	2	2
Case 12	72	21	F	visual hallucination	sCJD	MM	Type 1 > 2	2	2	2	2
Case 13	94	90	F	hearing disturbance	V180I	MV	Type 2 equivalence	2	2	2	2
Case 14	93	46	F	communication disturbance	V180I	MM	Type 2 equivalence	2	2	2	2
Case 15	70	19	M	dementia	M232R	MM	Type 1 equivalence	2	2	2	2
Case 16	74	7	M	dementia	dCJD	MM	Type 1 equivalence	2	2	2	2
Control 1	95		F		AD+DLB			0	0	0	0
Control 2	69		M		ALS			0	0	0	0
Control 3	53		F		ALS			0	0	0	0
Control 4	61		M		MSA			0	0	0	0

Typing of prion protein was performed on the basis of biochemical analysis of the frontal cortex. Diagnosis was performed on the basis of neuropathology and molecular biology analyses

Semi-quantification: 0 = none, 1 = positive and scattered staining, 2 = positive and consistent staining in both outer and inner plexiform layers

*Abbreviations: AAD* age at death, *M* male, *F* female, *sCJD* sporadic CJD, *dCJD* cadaveric dura matter graft CJD, *AD* Alzheimer’s disease, *DLB* dementia with Lewy body disease, *ALS* amyotrophic lateral sclerosis, *MSA* multiple system atrophy, *MM* methionine/ methionine, *VV* valine/valine, *−irs* -immunoreactive deposits

**Fig. 1:**
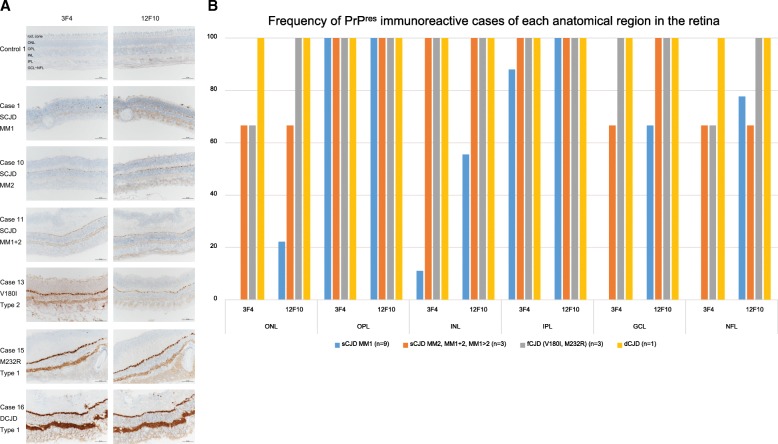
**a**. Representative images of PrP immunohistochemistry of retinas in Creutzfeldt-Jakob disease. 3F4 and 12F10 immunoreactive deposits are present in the OPL and IPL. 12F10 immunoreactive deposits stain more strongly than those of 3F4. In particular, cases of MM2, MM1 + 2, V180I, M232R, and dCJD show 3F4 and 12F10 immunoreactive fine deposits in the INL, ONL, GCL, and NFL. B. 3F4 and 12F10 immunoreactive deposits are consistently observed in the OPL and IPL. ONL: outer nuclear layers, OPL: outer plexiform layer, INL: inner nuclear layer, IPL: inner plexiform layer, GCL: ganglion cell layer, NFL: nerve fiber layer. **b**. Frequency of PrP immunoreactivity of each anatomical region in the retina. Fine-dot PrPres-irs staining was occasionally observed in the INL, ONL, and NFL. Staining was more consistent in cases of MM2, fCJD, and dCJD

In all CJD cases, 3F4 and 12F10-irs were consistently and clearly observed in the OPL and IPL of the neural retina (Fig. 1a). In our series, 12F10-irs staining was stronger than 3F4-irs. PrP^res^-irs staining exhibited granular and fine synaptic patterns in the OPL and IPL, respectively. Although PrP^res^-irs staining was always present in both OPL and IPL, PrP^res^-irs staining was stronger in sCJD (MM2), fCJD, and dCJD cases than in sCJD (MM1) cases (Fig. 1a).

In some instances, fine-dot PrP^res^-irs staining was observed in the INL, ONL, GCL and NFL. More consistent findings were observed in sCJD (MM2), fCJD, and dCJD cases (Fig. 1b). No PrP^res^-irs staining was present in the photoreceptor cell layer. In addition, there was no amyloid-beta (4G8), p-tau (AT8), p-synuclein, or TDP-43-irs staining in the retina. No PrP^res^-irs staining was observed in retinas from control cases. There was no clear PrP^res^-irs staining in the optic nerves.

Clinical characteristics, such as age at onset, duration, gender, and initial presentation were not associated with the presence or absence of PrP^res^-irs in the retina. Our methodology analyzing the posterior portion of the eye ball accurately reflected the pathologic condition of the neural retina in prion diseases. Indeed, a previous study showed that PrP^res^-irs were not prominent in the anterior portion of the neural retina [2].

Our study is the first to describe PrP^res^-irs within the retina in a series of cases of sCJD (MM1), sCJD (MM2, MM1 + 2), fCJD, and dCJD. Although we did not quantitatively evaluate the amount of PrP^res^ in each case, PrP^res^-irs in the OPL and IPL may be more prominent in fCJD and dCJD cases. In addition, PrP^res^-irs were occasionally observed within layers of neural retina other than the OPL and IPL.

Protease-sensitive normal cellular PrP was identified in the neural retina of healthy controls [1]. Because no 3F4-irs and 12F10-irs were present in control cases, we suspect that 3F4-irs and 12F10-irs in the retina of the present cases reflect PrP^res^ accumulation. Head et al. performed a detailed analysis of PrP^res^ in the neural retina [1, 2]; they found that PrP^res^-irs were present in the OPL (granular pattern) and IPL (synaptic pattern) in one case of sCJD (MM1) and two cases of variant CJD. PrP^res^ was reported as less detectable in the neural retina of sCJD (MM1) [2]. Another study reported the presence of PrP^res^ in the neural retina in vCJD, but not in sCJD, by Western blotting analysis [6]. In Gerstmann–Sträussler–Scheinker disease (F198S), PrP deposits were found in the inner portion of the OPL [7]. We suspect that the type of prion disease is associated with the pattern and severity of PrP^res^-irs in the neural retina. There was no clear association between the clinical duration and PrP^res^-irs in the neural retina.

The present study has some limitations. The sample sizes were small, except for cases of sCJD (MM1). This study was able to describe PrP^res^ staining in the OPL and IPL; thus, PrP^res^ must be analyzed in other layers of the retina with another methodology, such as laser micro-dissection or biochemical analysis, because PrP^res^-irs staining in other layers was very weak. In the future, specific eye examinations may become a potential biomarker for the clinical diagnosis of prion diseases, similar to potential clinical diagnosis of AD by detection of amyloid-beta deposits in the retina [8]. In terms of infection protection, we need to understand PrP^res^ accumulation in the neural retina is common findings even in atypical clinical form of sCJD (MM2, MM1 + 2) as well as fCJD and dCJD.

## Abbreviations

CJD: Creutzfeldt-Jakob disease
dCJD: Cadaveric dura matter graft Creutzfeldt-Jakob disease
fCJD: Familial Creutzfeldt-Jakob disease
GCL: Ganglion cell layer
INL: Inner nuclear layer
IPL: Inner plexiform layer
-irs: -Immunoreactive deposits
NFL: Nerve fiber layer
ONL: Outer nuclear layer
OPL: Outer plexiform layer
PrP: Prion protein
PrP^res^: Protease-resistant isoform of the prion protein
sCJD: Sporadic Creutzfeldt-Jakob disease

## References

[1] Head MW, Northcott V, Rennison K, Ritchie D, McCardle L, Bunn TJR, McLennan NF, Ironside JW, Tullo AB, Bonshek RE (2003) Prion protein accumulation in eyes of patients with sporadic and variant Creutzfeldt-Jakob disease. Invest Opthalmol Vis Sci:44. 10.1167/iovs.01-1273 10.1167/iovs.01-1273 12506094

[2] Head MW, Peden AH, Yull HM, Ritchie DL, Bonshek RE, Tullo AB, Ironside JW (2005). Abnormal prion protein in the retina of the most commonly occurring subtype of sporadic Creutzfeldt-Jakob disease. Br J Ophthalmol.

[3] Takao M, Kimura H, Mihara B (2017). How can we increase the number of autopsies for prion diseases? A model system in Japan. J Neurol Sci.

[4] Parchi P, Giese A, Capellari S, Brown P, Schulz-Schaeffer W, Windl O, Zerr I, Budka H, Kopp N, Piccardo P, Poser S, Rojiani A, Streichemberger N, Julien J, Vital C, Ghetti B, Gambetti P, Kretzschmar H (1999). Classification of sporadic Creutzfeldt-Jakob disease based on molecular and phenotypic analysis of 300 subjects. Ann Neurol.

[5] Parchi P, Strammiello R, Notari S, Giese A, Langeveld JP, Ladogana A, Zerr I, Roncaroli F, Cras P, Ghetti B, Pocchiari M, Kretzschmar H, Capellari S (2009). Incidence and spectrum of sporadic Creutzfeldt-Jakob disease variants with mixed phenotype and co-occurrence of PrPSc types: an updated classification. Acta Neuropathol.

[6] Wadsworth JD, Joiner S, Hill AF, Campbell TA, Desbruslais M, Luthert PJ, Collinge J (2001). Tissue distribution of protease resistant prion protein in variant Creutzfeldt-Jakob disease using a highly sensitive immunoblotting assay. Lancet.

[7] Head MW, Ironside JW, Ghetti B, Jeffrey M, Piccardo P, Will R, G. (2015) Prion diseases. In: Love S, Perry A, Ironside JW, Budka H (eds) Greenfield’s neuropathology 9th edn. CRC Press, City, pp 1016–1086

[8] Koronyo Y, Biggs D, Barron E, Boyer DS, Pearlman JA, Au WJ, Kile SJ, Blanco A, Fuchs D-T, Ashfaq A, Frautschy S, Cole GM, Miller CA, Hinton DR, Verdooner SR, Black KL, Koronyo-Hamaoui M (2017). Retinal amyloid pathology and proof-of-concept imaging trial in Alzheimer’s disease. JCI Insight.

